# The 4th Annual Ontario Thoracic Cancer Conference at Niagara-on-the-Lake

**Published:** 2009-09

**Authors:** Y.C. Ung, E. Yu, R. Malthaner, R. Burkes, P. Ellis, G. Goss, H. Solow, S. Irvine, S. Laffan

**Affiliations:** * Department of Radiation Oncology, Odette Cancer Centre, University of Toronto, Toronto, ON; † Division of Radiation Oncology, London Health Sciences Centre, University of Western Ontario, London, ON; ‡ Division of Thoracic Surgery, London Health Sciences Centre, University of Western Ontario, London, ON; § Mount Sinai Hospital, Princess Margaret Hospital, University Health Network, University of Toronto, Toronto, ON; || Department of Oncology and Clinical Epidemiology and Biostatistics, Juravinski Cancer Centre, McMaster University, Hamilton, ON; # The Ottawa Hospital Cancer Centre, University of Ottawa, Ottawa, ON; ** Markham Stouffville Hospital, Markham, ON; †† Continuing Health Sciences Education, McMaster University, Hamilton, ON

**Keywords:** Lung cancer, esophageal cancer, molecular targeted therapies

## Abstract

The 4th annual Ontario Thoracic Cancer Conference at Niagara-on-the-Lake focused on the themes of innovations in the management of lung cancer, controversies in the management of esophageal cancer, and molecular targeted therapies in lung cancer. This conference summary highlights the presentations and provides clinicians with a referenced update on these topics.

## 1. INTRODUCTION

The 4th annual Ontario Thoracic Cancer Conference was held at Niagara-on-the-Lake, April 17–19, 2009, bringing together health care professionals interested in thoracic oncology in the province of Ontario. Attendees at this conference spanned the disciplines of surgical, radiation, and medical oncology, respirology, pathology, nursing, support services, and radiation therapy. Advocates for lung cancer patients were also in attendance. For the first time this year, a session on the management of esophageal cancer was presented, as were a session on new innovations in radiation therapy and an update on molecular targeted therapy. A poster session highlighted research work being done by trainees, whose abstracts are published in the Appendix to this report.

## 2. HIGHLIGHTS

This year’s meeting highlighted three themes:

- Innovations in the management of lung cancer
- Controversies in the management of esophageal cancer
- Molecular targeted therapies for lung cancer

### 2.1 Innovations in Lung Cancer Management

#### 2.1.1 Radiation Therapy

Professor Jake Van Dyk, from the University of Western Ontario and the London Health Sciences Centre, delivered the first keynote address, “New Advances in Radiation Therapy for Non-Small Cell Lung Cancer” (nsclc).

Three major problems are encountered in the treatment of nsclc:

- Accurate delineation of the target
- Precision delivery of high-dose radiation to the target
- Minimization of radiation exposure to surrounding normal critical tissues—for example, normal lung and esophagus

The potential solutions for these problems involve better imaging by incorporating positron-emission tomography (pet) for accurate localization and by avoiding geometric misses 1–3, by using image-guided radiation therapy 4 for dose escalation and tumour adaptive changes to improve local control 5,6, and by minimizing collateral damage to critical tissues.

Innovative new radiation delivery treatment systems that include the use of tomotherapy 7, robotic radiosurgery, stereotactic body radiation treatment, and magnetic resonance–guided radiation have been evaluated. The use of respiratory gating methods to minimize exposure of normal lung tissue is important in the development of these new, highly conformal radiation techniques, so as to reduce the risk of radiation pneumonitis as the radiation dose is escalated beyond traditional levels.

Dr. Stewart Gaede, from the London Health Sciences Centre, spoke on “Respiratory Gating in Lung Cancer Applications, Including 4D CT–Based Treatment Planning.” Respiratory management techniques that include tumour tracking methods, tumour immobilization, breath-hold methods, and respiratory gating were reviewed. At the London Regional Cancer Centre, 96 patients have been treated using respiratory-gated radiotherapy with either liver metastases or lung tumours. Based on evaluation of dose–volume histogram parameters, respiratory gating reduces the amounts of normal lung and liver that receive a significant dose. However, optimal techniques are still being investigated to correlate the use of external marker motion with internal tumour or organ motion 8. Despite the uncertainties of dose distribution and organ tracking, the use of respiratory gating is a promising strategy to aid in dose escalation, in the avoidance of critical structures influenced by respiration, and in the delivery of intensity-modulated radiation treatments.

#### 2.1.2 Reducing Wait Times

Dr. Carol Sawka, Vice President, Clinical Programs and Quality Initiatives, Cancer Care Ontario (cco), gave the second keynote address on “Access to Cancer Services in Ontario: A Progress Report.”

Access to care can be defined as “equitable and timely access to appropriate care when needed.” The patient journey from onset of symptoms to treatment is influenced by many factors, which include the type and stage of cancer, the treatment and services selected by the physician, and patient choices for therapy. Cancer Care Ontario is dedicated to improving the patient experience, and the organization uses a number of strategies to improve the performance of cancer services. It has created the Cancer System Quality Index (csqi) 9, a Web-based public reporting tool that serves as a system-wide monitor tracking the quality and consistency of key cancer services that span the spectrum from prevention to end-of-life care. The csqi has about 30 key indicators, and each indicator is a specific measure of progress against one of six goals that help focus efforts to improve the cancer system in Ontario. One of the key indicators is wait times, whose principle is that wait time targets should be based on the biologic behaviour of the cancer. Wait times for surgical and radiation therapy are improving, but access to systemic therapy still needs improvement. The Lung Cancer Disease Pathway Management initiative at cco will focus on the patient journey to make improvements in the cancer system. It will bring together experts focused on lung cancer to evaluate the continuum of care, to map the patient journey, to evaluate the system’s performance, and to develop an integrated improvement program.

Methods to improve the performance of the system have included the development of thoracic diagnostic assessments units (daus) as presented by Dr. Matthew Kilmurry and Ms. Jennifer Parkins from the Grand River Regional Cancer Centre.

The dau was a joint venture between the regional cancer centre and its two host hospitals in the Waterloo–Wellington Local Health Integration Network (lhin). The lhin’s lung cancer patients have a long wait time and uncoordinated care pathways for referrals and diagnostic imaging. The dau provides timely access to diagnosis and treatment, interdisciplinary focused care, multidisciplinary case conferencing, and implementation of evidence-based care. The most common diagnostic test ordered is a computed tomography (ct)–guided biopsy, followed by bronchoscopy, mediastinoscopy, and pet imaging. To reduce the wait time from ct to diagnosis, pre-booked slots were made available in medical imaging. As a result, wait time was reduced to 27 days from 74 days, which parallels the initial experience of the Time to Treat initiative at the Toronto East General Hospital 10. Future plans include integrating the dau into the surgical oncology program, expanding the nursing role, and further engaging regional physicians. Cancer Care Ontario has set up a guideline on the establishment of diagnostic programs 11.

A diagnostic test commonly requested through the dau is pet imaging. An update on the role of pet in staging and managing lung cancer was presented by Dr. Yee Ung, from the Odette Cancer Centre. In lung cancer, pet imaging shows high sensitivity and specificity over conventional imaging, a finding that has been systematically reviewed^12^. More recently, two clinical trials in lung cancer by the Ontario Clinical Oncology Group have shown the utility of pet for staging the mediastinum in early-stage resectable lung cancer 13 and in selecting appropriate locally advanced lung cancer patients for aggressive combined-modality therapy 14. As a result of these clinical trials, lung cancer patients in Ontario now have access to pet imaging as part of their care when they fit the foregoing criteria.

#### 2.1.3 Interdisciplinary Care

The patient journey for lung cancer involves interaction with many disciplines, and there is an expanding role for the advanced practice nurse (apn), as presented by Ms. Lorraine Martelli–Reid, an apn from the Juravinski Cancer Centre.

The roles of the apn span the spectrum from clinical care to education, research, and organizational leadership. A retrospective review at the Juravinski Cancer Centre (jcc) of patients undergoing postoperative adjuvant chemotherapy with cisplatin and vinorelbine in the National Cancer Institute of Canada (ncic) Clinical Trials Group (ctg) br.10 15 trial showed an absolute survival difference of 15%. However, the chemotherapy regimen is difficult to complete: only 50% of patients on the ncic br.10 clinical trial were able to complete all 4 cycles of chemotherapy. At the jcc, however, 84% of patients were able to complete the 4 cycles. Support from an apn is vital in helping to manage symptoms during chemotherapy and in providing education and counselling for the patients. A novel apn-led “Take a Breather Clinic” was established to help lung cancer patients with symptoms of dyspnea. Dyspnea had been identified as a significant symptom using the Edmonton Symptom Assessment System (esas).

Use of the esas as a common tool for assessment was initiated through the Provincial Palliative Care Integration Project (ppcip) funded by the Ministry of Health and Long-Term Care and cco. Dr. Jeff Myers, palliative physician and Toronto Central lhin lead for the project, indicated that the aim was to target all lung cancer and palliative care patients in the regional cancer centres and all palliative patients in the home setting. By using common tools that incorporate a symptom measurement scale (that is, the esas 16), symptom management guidelines for intervention, and a palliative performance scale, it would be possible to evaluate patient symptoms and to monitor progress through the course of a patient’s care. The success of the ppcip led to the next stage (that is, the Ontario Cancer Symptom Management Collaborative), which includes all cancer patients with participation of all regional cancer centres and community care access centres. This project has given a “voice” to the patient’s symptoms, which may or may not be usually discussed, and has provided a common language for communication between care providers.

### 2.2 Controversies in the Management of Esophageal Cancer

This year’s meeting explored selected issues in esophageal cancer. Adenocarcinomas of the esophagus and gastroesophageal junction are increasing in incidence, and squamous cell cancers are decreasing. The 5-year survival rates are poor in surgically resected patients, emphasizing the need for more effective adjunctive therapies. Dr. Jennifer Knox from the Princess Margaret Hospital reviewed the major clinical trials involving preoperative chemotherapy 17–19 or preoperative chemoradiation 20.

Preoperative chemoradiation for adenocarcinomas improves overall survival and achieves higher pathologic complete response rates than are seen with chemotherapy alone, but the regimen is more toxic. Improvement in overall survival is less certain with preoperative chemoradiation in squamous cell carcinomas, but local control is improved over that with surgery alone. The ability to evaluate response to neo-adjuvant therapy would be useful for prognostication. The municon trial 21 evaluated the strategy of using pet to determine the length of preoperative chemotherapy before surgery in locally advanced gastroesophageal junction cancers. In that trial, patients underwent a pet scan at baseline and then proceeded to neoadjuvant chemotherapy with cisplatin and 5-fluorouracil. After 2 weeks of therapy, a re-evaluation pet scan separated the metabolic non-responders (less than 35% decrease in standardized uptake value) from the responders. The non-responders by pet proceeded to surgery immediately; the responders continued the rest of their chemotherapy to 12 weeks before receiving surgery. Responders by pet had a 96% R0 resection rate and a 58% major pathologic response (defined as less than 10% residual tumour).

The use of pet in radiation treatment planning was evaluated by Dr. Danny Vesprini and colleagues from the Odette Cancer Centre. Their study evaluated the effect of the addition of fused pet–ct imaging over ct alone in the identification of the gross tumour volume (gtv) in patients with esophageal cancer 22. Ten patients with esophageal cancer underwent pet and ct imaging in radiation treatment position, and the resulting image sets were fused. Six radiation oncologists independently contoured the gtv using ct data alone, supplemented with standardized clinical and diagnostic imaging information. The same radiation oncologists then contoured the gtv using the co-registered pet–ct images. The standard deviation of the gtv length and volume were used a measure of inter-observer and intra-observer variation. The average observer agreement index using pet–ct was 72.7% as compared with 69.1% using ct alone. The pet–ct significantly improved both inter-observer and intra-observer variability in the identification of the primary gtv.

### 2.3 Molecular Targeted Therapies for Lung Cancer

The third keynote address on “Current Perspectives in the Treatment of Advanced Non-Small Cell Lung Cancer” was given by Dr. Natasha Leighl, from the Princess Margaret Hospital.

As the biology of tumour progression becomes better understood, newer targets for biologic therapies will become available for clinical trials. Currently, a wealth of molecular targeted therapies are under investigation in nsclc. The most promising new therapies target either the vascular endothelial growth factor (vegf) 23 or the epidermal growth factor receptor (egfr) 24. Two important randomized phase iii clinical trials evaluated the addition of bevacizumab to standard chemotherapy as compared with standard chemotherapy alone in advanced-stage (iiib/iv) and recurrent nsclc. The Eastern Cooperative Oncology Group E4599 trial 25 and the avail trial 26 showed improved progression-free survival for their bevacizumab arms. In the E4599 trial, overall median survival also improved to 12.3 months from 10.3 months [hazard ratio (hr): 0.79; *p* = 0.003], and the adenocarcinoma subgroup had a more significant improvement in overall median survival to 14.2 months (hr: 0.69). The avail trial did not demonstrate a survival benefit. The incidence of grade 3 or more serious adverse events on these trials was low, ranging from 0.3% to 9% for bleeding, hypertension, proteinuria, febrile neutropenia, and arterial thrombosis.

A promising vegf small-molecule inhibitor, cediranib, was evaluated by the ncic ctg in a phase ii/iii study design—the ncic br.24 trial. Patients were randomized to carboplatin and paclitaxel with cediranib or a placebo; the cediranib arm had an increased response rate of 38% as compared with 16% (hr: 0.77) 27, but some toxic deaths from dehydration and diarrhea occurred. The follow-up trial, ncic br.29 will use a lower dose of cediranib to reduce the occurrence of side effects.

The flex trial evaluated the use of cisplatin and vinorelbine with or without cetuximab (a monoclonal antibody against egfr) in first-line treatment of stage iiib/iv nsclc. The cetuximab arm had a median overall survival of 11.3 months as compared with 10.1 months, and a 1-year survival of 47% as compared with 42% (hr: 0.871; *p* = 0.044) 28. Patients who developed an early acne-like rash experienced a median overall survival of 15 months.

A current controversy is the role for maintenance therapy after completion of first-line treatment with a platinum doublet. In a trial by Ciuleanu *et al.* 29 of maintenance after completion of platinum chemotherapy, patients were randomized to either pemetrexed or placebo (2:1 randomization), resulting in a median progression-free survival of 4.3 months as compared with 2.6 months (hr: 0.502; *p* < 0.00001), and an improvement in overall survival to 13.4 months as compared with 10.6 months (hr: 0.79; *p* = 0.012), with a significant difference of 14.4 months as compared with 9.4 months (*p* = 0.0025) in non-squamous histology. Similar results were seen for the use of targeted therapies for maintenance on the saturn 30 and atlas 31 trials that used erlotinib, or bevacizumab with or without erlotinib, although survival data are pending.

## 3. THE GREAT DEBATES

Three issues were debated at this year’s meeting:

- Stereotactic body radiation therapy (sbrt) compared with surgery for T1N0 lung cancer
- Preoperative compared with postoperative chemoradiation for esophageal cancer
- Endoscopic mucosal resection compared with surgery for esophageal cancer

### 3.1 SBRT Versus Surgery for T1N0 NSCLC

Dr. Patrick Cheung, Odette Cancer Centre, debated Dr. Richard Inculet, London Health Sciences Centre in the first debate of radiation versus surgery for early-stage nsclc.

A comparison of current outcomes in patients treated using sbrt with those in patients undergoing surgery for stage i nsclc are limited by the accuracy of staging. Patients referred for radiation often have significant medical comorbidities that preclude surgical resection, and they are often clinically staged where surgical candidates are pathologically staged. In addition, the radiation dose used to control early-stage nsclc is very important: The dose given must be effective enough to eradicate small lung cancers.

In a large retrospective multi-institutional study of sbrt using biologically effective doses of radiation for stage i nsclc, a 5-year survival rate of 53.9% was achieved, and in the subset of operable lung patients, the survival increased to 70.8% 32. The toxicities associated with sbrt for peripheral locations are minimal; they include radiation pneumonitis (5.4%), mild dermatitis (1.2%), and rib fracture (1.6%). Centrally located lesions may carry a higher risk of bronchial stenosis with lung collapse, and current clinical trials are evaluating the safety of treating central lesions with sbrt. The major concern with sbrt is the effect of radiation on patients with poor pulmonary function. However, an analysis of 70 medically inoperable stage i nsclc patients with poor baseline pulmonary function did not predict for decreased survival or decreased pulmonary function after treatment 33.

Surgical resection is still the standard of care for resectable early-stage nsclc. Innovations with minimally invasive surgical techniques—that is, video-assisted thoracoscopic surgery (vats)—have reduced surgical morbidity. In selected cases, outcomes may be better than those with standard lobectomy 34. The safety and efficacy of vats lobectomy compared with open lobectomy have been systematically reviewed, and no statistically significant differences were observed in terms of postoperative prolonged air leak, arrhythmia, pneumonia, mortality, or risk of locoregional recurrence 35. Today, patients that might not have been considered for open lobectomy may therefore, with vats, still be surgical candidates.

The choice of sbrt or surgery for stage i nsclc will be multifactorial, but appropriately selected patients will do well with either option.

### 3.2 Preoperative Versus Postoperative Chemoradiation for Esophageal Cancer

Dr. Rebecca Wong, Princess Margaret Hospital, debated Dr. Richard Malthaner, London Health Sciences Centre, in the second debate on esophageal cancer.

Surgery alone is insufficient treatment for resectable, but locally advanced, cancers of the esophagus because locoregional and distant recurrence rates are significant. Therefore using either preoperative or postoperative therapy may be useful in improving outcomes. The advantage of using a preoperative approach are these:

- Tumour downstaging can occur before surgical resection.
- Radiation target volumes are smaller.
- Perioperative morbidity is less.
- Radiation dose is more effective in an undisturbed tumour.

In esophageal cancer, 10 randomized controlled clinical trials have involved 1209 patients. The hr for all-cause mortality was 0.81 for neoadjuvant chemoradiotherapy as compared with surgery alone, corresponding to a 13% absolute difference in survival at 2 years favouring neoadjuvant chemoradiation 20.

Postoperative chemoradiotherapy holds these advantages:

- Postoperative adjuvant therapy can be tailored using the accurate stage.
- Unnecessary treatment of early-stage esophageal cancer is avoided.
- Surgery is better tolerated.
- Immediate improvement is achieved in the major presenting symptom, dysphagia.

No randomized controlled trials have compared postoperative chemoradiation with surgery alone, and none have compared preoperative chemoradiation with postoperative chemoradiation. At the London Regional Cancer Centre, a retrospective review of patients with lymph-node-positive disease who, after surgical resection, were given postoperative chemoradiation showed that the postoperative treatment, as compared with no treatment, was associated with significantly longer survival 36. A definitive clinical trial comparing preoperative with postoperative chemoradiation would be useful in determining the precise benefit in terms of survival and quality of life.

### 3.3 Endoscopic Mucosal Resection Versus Surgery for Esophageal Cancer

Dr. Norman Marcon, St. Michael’s Hospital, debated Dr. Richard Inculet, London Health Sciences Centre, in the third debate on the treatment of early esophageal cancer.

The dilemmas faced in the treatment of high-grade dysplasia and intramucosal adenocarcinoma 37 include

- the confidence of the pathologic diagnosis,
- the malignant risk of the lesion,
- the completeness of the resection,
- the morbidity and mortality of the treatment, and
- the eradication of the disease.

Endoscopic therapy can be either endoscopic mucosal resection or endoscopic submucosal dissection (basically removing the mucosal tissue down to and including the submucosa). For endoscopic resection to be successful, there must be accurate staging of the disease, a low failure rate, an accurate method of surveillance, good functional results post treatment, and an effective way to deal with the underlying cause—that is, gastroesophageal reflux disease 38. For surgical resection to be successful, there must be a low complication rate, a reasonable functional result, and a high curative potential 39. Both treatment options are suitable, depending on patient compliance, disease characteristics, extent of disease, and expertise of the treating physician.

## 4. POSTER PRESENTATIONS

Research by the medical trainees was highlighted in the poster presentations. The abstract review committee selected two posters for oral presentation. The first, by Dr. Meredith Giuliani, was titled “Prophylactic Cranial Irradiation Utilization Rates in Limited-Stage Small-Cell Lung Cancer.” The second, by Dr. Jeffrey Cao, was a “Systematic Review of the Cost-Effectiveness of PET in Staging of Non- Small-Cell Lung Cancer and Management of Solitary Pulmonary Nodules.” The abstracts are published in the Appendix to this report.

## 5. SUMMARY

The Ontario Thoracic Cancer Conference continues to bring together people interested in the management of patients with thoracic malignancies. It remains an excellent forum to foster research and wide multidisciplinary interaction. We extend our thanks to all who made this meeting such a success, including our sponsors, Astra Zeneca and Lilly (platinum level), Olympus (gold level), and Boehringer Ingelheim (silver level).

## APPENDIX: Abstracts Presented at the 4th Ontario Thoracic Cancer Conference

### 10 Years of VCRT: An Analysis of a Novel Protocol of Combined Concurrent Chemoradiation in Unresectable Non-Small-Cell Lung Cancer

Waters E, Rodrigues G, Vincent M, Dingle B. London Regional Cancer Centre, University of Western Ontario, London, Ontario.

#### Background and Objectives

The London Regional Cancer Program (lrcp) employs a unique schedule of concurrent chemoradiation, termed vcrt (vinblastine, cisplatin, radiation therapy), for the treatment of unresectable stage iiia and iiib non-small-cell lung cancer (nsclc). The protocol consists of 100 mg/m^2^ cisplatin and 6 mg/m^2^ vinblastine (reduced to 4.2 mg/m^2^ during cycles 3 and 4), split over days 1–3, given once every 3 weeks for 4 cycles, with 60 Gy of concurrent radiation over 6 weeks during cycles 3 and 4.

The objective of the present study was to determine overall survival and to characterize outcomes of patients treated with vcrt.

#### Methods

This retrospective analysis, with a focus on overall survival and toxicities, reports a cohort of 294 patients who underwent vcrt at the lrcp between 1996 and 2006.

#### Results

The overall 5-year survival, determined using Kaplan–Meier methodology, was 19.8%, and the median survival duration was 18.2 months. Reported grades 3 and 4 toxicities included neutropenia (39%), anemia (10%), pneumonitis (1%), and esophagitis (3%). Log-rank tests demonstrated significant differences in survival between groups of patients for completion surgery, use of radiation therapy, and cisplatin dose. Similarly, univariate Cox regression showed that completion surgery, use of radiation therapy, cisplatin dose, and vinblastine dose were significant factors in the survival of all stage iii nsclc patients treated with vcrt.

#### Discussion and Conclusions

This retrospective analysis reveals an overall survival comparable to that of other current combined chemoradiation protocols. The success of the vcrt protocol seems to be dose-dependent.

### Characteristics of Lung Cancer at a Regional Cancer Centre

Alam Y, Zhao Y. Windsor Regional Cancer Centre, Windsor, Ontario; Schulich School of Medicine, University of Western Ontario, London, Ontario.

#### Background and Objectives

Lung cancer is strongly correlated with cigarette smoking, although other environmental and genetic components may also play a role. There have been public concerns in Southwestern Ontario that environmental and occupational exposures are resulting in a higher lung cancer rate.

The present study aimed to further characterize the smoking habits, histology subtypes, stage at diagnosis, and 5-year survival rates in newly diagnosed lung cancer patients in the Southwestern Ontario region in 2003.

#### Methods

We retrospectively reviewed all incident lung cancer patients seen at the Windsor Regional Cancer Centre (wrcc) in 2003. Data were collected from the e-chart database used at the centre. Age, presenting symptoms, histology, initial clinical staging, and 5-year survival were recorded.

#### Results

In 2003, 189 new lung cancer cases were seen at the wrcc. In 93.65% of the cases, the patient was 50 years of age or older. In 93.12% of the cases, the patient was a smoker or ex-smoker. Weight loss, cough, and dyspnea were the most common presenting symptoms. Non-small-cell lung cancer (nsclc) constituted 74.07% of the cases, of which 64% were stage iii or iv at initial diagnosis. The small-cell subtype accounted for 14.99% of the cases, and 61.76% of those patients had extensive disease at diagnosis. Survival at 5 years was 5.82%.

#### Discussion and Conclusions

More than 90% of newly diagnosed lung cancer patients from the Southwestern Ontario region in 2003 were smokers and 50 years of age or older. The histology subtypes and survival trends for these patients were similar to trends published elsewhere in the literature. Another review for 2008, to see if the characteristics have changed, would be interesting.

### Adjuvant Chemotherapy in Non-Small-Cell Lung Cancer—Boon or Bane?

Kuruvilla MS, Martelli-Reid L, Goffin JR, Arnold A, Ellis PM. Juravinski Cancer Centre, McMaster University, Hamilton, Ontario.

#### Background and Objectives

Published data support the use of cisplatin–vinorelbine doublet as adjuvant chemotherapy in completely resected non-small-cell lung cancer (nsclc). However, data also demonstrate a need for frequent dose reductions. Consequently, many centres across Canada have been reluctant to adopt this practice.

The primary objective of the present study was to assess the deliverability of this adjuvant regimen in patients with stage ib, ii, and iii nsclc within our center. Secondary objectives were to determine the tolerability and toxicity of this regimen.

#### Methods

We retrospectively reviewed patients with nsclc receiving adjuvant cisplatin–vinorelbine at the Juravinski Cancer Centre between January 2005 and September 2007. Demographics, total chemotherapy dose, treatment duration, and toxicity profiles were abstracted. Relative dose intensity (rdi) was calculated as a marker of deliverability.

#### Results

Adjuvant cisplatin–vinorelbine was administered to 41 patients. The median weekly dose intensity was 23.5 mg/m^2^ (range: 13.1–27.9 mg/m^2^) for cisplatin and 18.8 mg/m^2^ (range: 8.4–25.1 mg/m^2^) for vinorelbine. The median rdis for cisplatin and vinorelbine were 94% and 63%. Of the treated patients, 71% underwent all 4 cycles; 61% received all treatments of cisplatin, and 16%, all treatments of vinorelbine. Toxicities at grade 3 or higher included anemia (12%), neutropenia (63%), febrile neutropenia (7%), constipation (2%), and fatigue (18%). Blood transfusions were given to 24% of the patients, and 18% received erythropoiesis-stimulating agents. Less severe toxicities included peripheral neuropathy, ototoxicity, mucositis, vomiting, alopecia, and flare of surgical site pain.

#### Discussion and Conclusions

The deliverability of adjuvant cisplatin–vinorelbine, administered weekly for 16 weeks, was as good as or better than that in the anita trial. This finding helps to attenuate concerns about the tolerability of the regimen.

### Differences in *V*_20_ and Contoured Radiotherapy Treatment Volumes in Stage III Non-Small-Cell Lung Cancer with the Addition of Positron-Emission Tomography Imaging to Standard Computed Tomography Imaging

Chan E, Kiss A, Balogh J, Barbera L, Cheung P, Poon I, Spayne J, Ung YC. Odette Cancer Centre, Sunnybrook Health Sciences Centre, University of Toronto, Toronto, Ontario.

#### Background

Imaging by positron-emission tomography (pet) is increasingly used to stage and plan radiotherapy (rt) in patients with non-small-cell lung cancer (nsclc). The effect of this approach on rt volumes is not yet fully known. The present study evaluated differences in *V*_20_ (a predictor for radiation pneumonitis) and contoured rt volumes in pet-versus computed tomography (ct)–based plans for stage iii nsclc.

#### Methods

As part of their work-up, 18 patients underwent pet and ct. The rt volumes were initially contoured using the ct data alone. Contours were then modified by information from the pet imaging. Differences between the paired contours and *V*_20_ determinations for each patient were calculated.

#### Results

The average difference between the total contoured gross tumour volume for the pet and ct approaches was similar (1.13 cm^3^). In 5 of 18 patients, volume differences of more than 25 cm^3^ were observed. The average difference between the contoured planning target volumes for the two approaches was more varied (pet volumes were larger on average by 12.99 cm^3^). In 6 of 15 patients, volume differences of more than 50 cm^3^ were observed. The calculated *V*_20_ was similar between the two approaches (ct plans were larger on average by 0.19%) with a range of −2.8% to 4.5%. The range of *V*_20_ based on ct was 15.2% to 37.7%; on pet, it was 14.7% to 36.7%.

#### Conclusions

Overall, there appears to be only a small change between the contoured gtv and the planning target volume when using pet or ct imaging. However, in a proportion of patients, the contoured volumes are quite different. The ultimate effect of these differences will need to be validated by clinical outcomes.

### Inter-observer and Intra-observer Reliability for Lung Cancer Target Volume Delineation

Louie A, Rodrigues G, Gaede S. London Regional Cancer Centre, University of Western Ontario, London, Ontario.

#### Objectives

The purpose of the present study was to investigate inter- and intra-observer target volume delineation (tvd) error in the setting of four-dimensional (4D) computed tomography (ct) image data acquisition in thoracic tumours.

#### Methods

Six radiation oncologists contoured the primary and nodal gross tumour volume (gtv) of 10 lung tumours on the 10 respiratory phases of a 4D ct scan. The coefficient of variation (cov) and the percentage shared internal target volume (sitv) of the 6 physicians for each patient was used to assess inter- and intra-observer variability.

Analysis of variance was performed to assess differences in inter- and intra-physician variability based on patient case difficulty, respiratory phase, physician seniority, and physician observer.

#### Results

Inter-physician percentage sitv for primary tumour ranged from 31.1% to 83.3% [standard deviation (sd): 4.4%–15.8%] and from 16.4% to 66.8% (sd: 5.8%–21.1%) for nodes. Intra-physician sitv for primary tumour ranged from 59.6% to 72.7% (sd: 13.0%–23.9%), and from 28.3% to 57.0% (sd: 18.6%–34.2%) for nodes. Analysis of variance for covs found case difficulty (easy vs. difficult) to be significant for inter-physician primary tumour and intra-physician nodal disease delineation. Physician seniority, respiratory phase, and individual physician were not found to be significant for tvd error.

#### Conclusions

High observer variability in tvd continues to be a major source of error in the 4D ct era for lung cancer. Inter-physician variability appears to be the more significant source of this error than intra-physician variability. Development of measures to reduce inter- and intra-observer tvd variability are necessary to the delivery of high-quality radiotherapy.

### Stereotactic Body Radiotherapy for Inoperable Patients with Early Stage Non-Small-Cell Lung Cancer

Taremi M, Dahele M, Bezjak A. Princess Margaret Hospital, University Health Network, University of Toronto, Toronto, Ontario.

#### Background and Objectives

Stereotactic body radiotherapy (sbrt), a technique to deliver high-dose radiation in each fraction, is expected to provide high rates of local control.

#### Methods

Patients eligible for our lung sbrt Research Ethics Board–approved protocol included those with inoperable early-stage nsclc (T1/T2, N0, M0), and patients with a limited number of pulmonary metastases.

Two dose/fractionation (fr) schedules for peripheral tumours are 48 Gy/4 fr for T1 and 54–60 Gy/3 fr for T2 tumours. If the tumour is in proximity to midline structures, 60 Gy/8 fr or 50 Gy/10 fr is used. Toxicity and tumour response are assessed using Common Terminology Criteria for Adverse Events v.3 and the Response Evaluation Criteria in Solid Tumors criteria respectively.

#### Results

Between December 2004 and July 2008, 111 patients (median age: 72 years) were treated. The data for 96 patients with early-stage non-small-cell lung cancer (100 lesions) and a median follow-up of 16.4 months were analyzed for this report. In patients with at least 6 months of follow-up, we observed 42 partial responses, 35 complete responses, 10 stable disease, and 11 disease progression. Local failure occurred for 9 lesions, 5 of which were treated with 50 Gy/10 fr.

The estimated 3-year overall survival was 48% [95% confidence interval (ci): 32% to 62%]; cause-specific survival was 83% (95% ci: 72% to 94%). The most common acute toxicity was fatigue (42 patients). No patient had grade 4 or 5 toxicity.

#### Discussion and Conclusions

In early-stage nsclc, sbrt is an effective and well-tolerated treatment. However, careful patient selection, attention to planning and treatment delivery, and ongoing follow-up is needed to fully define the therapeutic ratio for this technique.

### Prophylactic Cranial Irradiation Utilization Rates in Limited-Stage Small-Cell Lung Cancer

Giuliani M, Hope A, Sun A, Ma C, Brade A, Cho JBC, Bezjak A. Princess Margaret Hospital, University Health Network, University of Toronto, Toronto, Ontario.

#### Background and Objectives

Prophylactic cranial irradiation (pci) improves survival in patients with limited-stage small-cell lung cancer (lssclc). The objective of the present audit was to assess the adoption of pci at our institution.

#### Methods

From 1997 to 2007, 796 patients were treated at Princess Margaret Hospital for sclc. Of these 796 patients, the 226 (28.4%) who received radical ls-sclc treatment formed the basis of this project. Brain failure-free survival (ffs) was estimated by the Kaplan–Meier method, comparing patients treated with and without pci.

The pci uptake was determined, and for patients not receiving pci, the reason was recorded if it was available. The Fisher exact test was used to compare rates of pci use during 1997–2001 and 2002–2007.

#### Results

Median follow-up was 15.5 months (range: 2–130 months). From 1997 to 2007, 55.3% (*n* = 125) of radically treated ls-sclc patients received pci. Brain ffs at 6, 12, and 24 months was 94.3%, 69.3%, and 32.0% respectively for patients who did not receive pci and 100.0%, 94.6%, and 76.8% respectively for patients who did receive that treatment (*p* < 0.0001). A nonsignificant increase in pci uptake, 51.7% to 59.1% (*p* = 0.29), occurred from 1997–2001 to 2002–2007. The most common reasons for not receiving pci were patient refusal and disease progression.

#### Discussion and Conclusions

Prophylactic cranial irradiation significantly improves brain ffs; only half of ls-sclc patients at our institution received pci. Uptake of pci has not significantly increased in recent years. A thorough exploration of patient concerns regarding pci may increase the uptake rate.

### Systematic Review of the Cost-Effectiveness of Positron-Emission Tomography in the Staging of Non-Small-Cell Lung Cancer and the Management of Solitary Pulmonary Nodules

Cao JQ, Rodrigues GB. London Health Sciences Centre, University of Western Ontario, London, Ontario.

#### Background and Objective

This systematic review describes the cost-effectiveness of positron-emission tomography (pet) imaging in the staging of nsclc and the management of solitary pulmonary nodules (spns).

#### Methods

We conducted systematic literature searches in the medline/Premedline, embase, and U.K. National Health Service databases. Measurement of study quality was assessed by the validated Quality of Health Economic Studies (qhes) instrument. Studies with a qhes score below 75 were excluded. Characteristics including study methodology, assumptions, and cost effectiveness metrics [incremental cost-effectiveness ratio—icer—based on life-years saved and average cost savings per patient (acsp)] were abstracted. Descriptive statistics were generated with cost amounts converted to a common inflation-adjusted 2007 U.S. dollar.

#### Results

The 20 studies that met all inclusion criteria, including acceptable qhes scores as determined by two reviewers (mean: 87.8), were based on the national health insurance payer perspective of 8 different countries. Investigations assessed the spn scenario (*n* = 8), the staging scenario (*n* = 11), and the spn and staging scenarios (*n* = 1) together. Mean assumed cost of pet scanning was $1267 (range: $769–$2580) in these studies. Median icers for spn and staging were $2039 (range: $181–$3927) and $4037 (range: $527–$32618) respectively. Median acsps for spn and staging were $518 (range: $66–$1480) and $1390 (range: $143–1633) respectively.

#### Conclusions

Reported cost-effectiveness metrics are highly variable and depend on input variables and assumptions including: cost, disease prevalence, diagnostic operating characteristics, the diagnostic strategies assessed, and the methodologies used. Despite this variation, these studies have consistently concluded that pet has favourable cost-effectiveness characteristics as compared with non-pet strategies.

### The Impact of Positive Surgical Resection Margins and Short Disease-Free Interval on Survival Following Relapse After Esophagectomy and Adjuvant Chemoradiation Therapy in High-Risk Esophageal Cancer

Yu E, Tai P, Malthaner R, Stitt L, Rodrigues G, Dar R, Yaremko B, Younus J, Sanatani M, Vincent M, Dingle B, Fortin D, Inculet R. London Health Science Centre, University of Western Ontario, London, Ontario, and Allan Blair Cancer Center, Regina, Saskatchewan.

#### Objective

This study investigated the effect of resection margin status and time interval to relapse on outcomes in high-risk esophageal cancer patients.

#### Patients and Methods

During 1989–1999, we followed high-risk resected esophageal cancer patients who completed postoperative chemoradiation therapy. Adjuvant chemotherapy consisted of 4 cycles of epirubicin, cisplatin, and 5-flurouracil (ecf), with epirubicin omitted during the radiation therapy (rt) phase. Total rt dose was 45–60 Gy at 1.8–2.0 Gy per fraction. Patients who relapsed after a disease-free interval of more than 3 months were treated with palliative chemoradiation when appropriate. Patients who relapsed after a disease-free interval of 3 months or less were treated with best supportive care. Logistic regression and log-rank tests were used for post-recurrence survival analysis.

#### Results

Of the 69 patients treated with adjuvant chemoradiation post-esophagectomy, 46 patients experienced recurrence. Median time to relapse was 28 months (range: 0.1–40 months). Median age of relapse patients was 61 years (range: 37–82 years). There were 42 male, 44 node-positive, 31 adenocarcinoma, and 33 clear resection margin post-esophagectomy patients. Median follow-up after recurrence was 30.5 months (range: 1.3–100 months). The median post-recurrence overall survival duration was 5.8 months, with overall survival rates of 20%, 10%, and 5% at 12 months, 24 months, and 36 months respectively. Of the prognostic factors analyzed, only resection margin status and time interval to recurrence were statistically significant for patient outcome in univariate and multivariate analysis.

#### Conclusions

Surgical resection margin status and time interval to disease relapse were independent prognostic factors for patient outcome.

The opinions expressed in the abstracts are those of the authors and are not to be construed as the opinion of the publisher (Multimed Inc.), or the organizers of the 4th Annual Ontario Thoracic Cancer Conference.

Although the publisher (Multimed Inc.) has made every effort to accurately reproduce the abstracts, Multimed Inc. and the organizers of the 4th Annual Ontario Thoracic Cancer Conference assume no responsibility and/or liability for any errors and/or omissions in any abstract published.
